# A review of the hormones involved in the endocrine dysfunctions of polycystic ovary syndrome and their interactions

**DOI:** 10.3389/fendo.2022.1017468

**Published:** 2022-11-15

**Authors:** Rebecca H. K. Emanuel, Josh Roberts, Paul D. Docherty, Helen Lunt, Rebecca E. Campbell, Knut Möller

**Affiliations:** ^1^ Department of Mechanical Engineering, University of Canterbury, Christchurch, New Zealand; ^2^ Institute of Technical Medicine, Furtwangen University, Villingen-Schwenningen, Germany; ^3^ Diabetes Services, Te Whatu Ora Waitaha Canterbury, Canterbury, New Zealand; ^4^ Department of Medicine, University of Otago, Christchurch, New Zealand; ^5^ School of Biomedical Sciences, Department of Physiology, Centre for Neuroendocrinology, University of Otago, Dunedin, New Zealand

**Keywords:** PCOS, hormones, endocrine, reproductive, metabolic

## Abstract

Polycystic ovary syndrome (PCOS) affects up to 20% of women but remains poorly understood. It is a heterogeneous condition with many potential comorbidities. This review offers an overview of the dysregulation of the reproductive and metabolic systems associated with PCOS. Review of the literature informed the development of a comprehensive summarizing ‘wiring’ diagram of PCOS-related features. This review provides a justification for each diagram aspect from the relevant academic literature, and explores the interactions between the hypothalamus, ovarian follicles, adipose tissue, reproductive hormones and other organ systems. The diagram will provide an efficient and useful tool for those researching and treating PCOS to understand the current state of knowledge on the complexity and variability of PCOS.

## 1 Introduction

Polycystic ovary syndrome (PCOS) is a heterogeneous condition that is reported to affect between 8% to 20% of women (1, 2). Currently, PCOS is diagnosed with the Rotterdam diagnostic criteria (3). To diagnose PCOS with the Rotterdam criteria, two of the following criteria must be observed: evidence of clinical and/or biochemical hyperandrogenism, evidence of oligo-ovulation and/or anovulation, or evidence of polycystic ovarian morphology through ultrasound (1). PCOS has a number of significant comorbidities associated with it, including type 2 diabetes (T2DM) (4, 5), cardiovascular disease (6), insulin resistance (7), obesity (8), infertility (6), pregnancy complications (9, 10), sleep disturbances (11), hypothyroidism (12), decreased mental health (13), and non-alcoholic fatty liver disease (NAFLD) (14).

**Figure 1:**
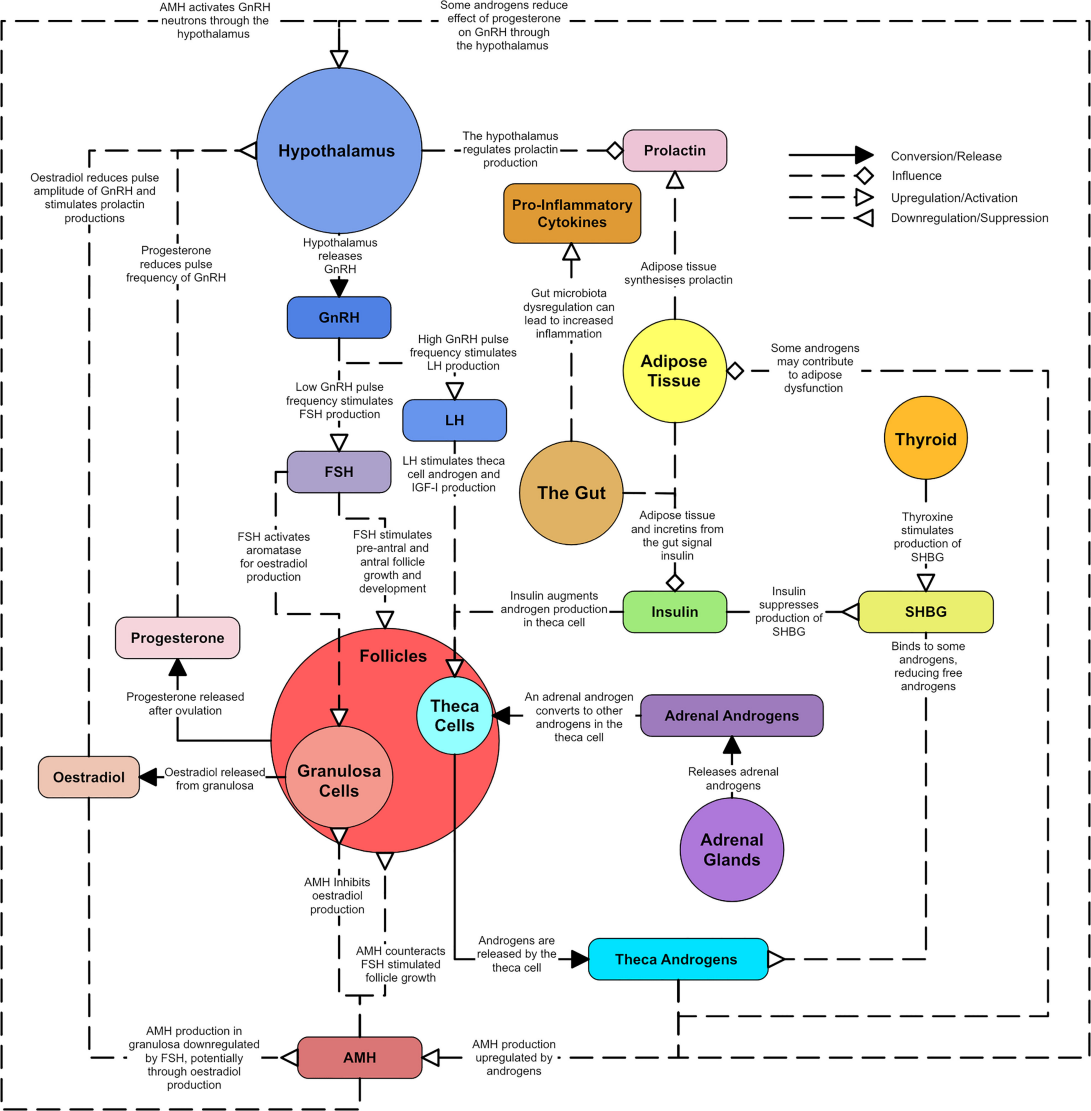
A simplified overview of the master diagram showing the key relationships and dysregulations of endocrine components in PCOS. Solid line arrows between features are used to indicated conversion or release. Dashed lines with diamond arrowhead connections are used to indicate influence. Dashed lines with arrowheads are used to indicate upregulation and dashed lines with reverse arrowheads are used to represent downregulation.

PCOS is a common metabolic and reproductive syndrome with heterogenous clinical presentations (‘syndromes’) that have no simple, single diagnostic or clinical management pathway. In addition there are multiple, poorly understood etiological factors that contribute to the heterogenous clinical expression of PCOS and these include genetic, environmental and hormonal factors. This paper focuses on a review of hormonal factors for the following reasons: There is an extensive body of research in this area but previous research has tended to focus on one hormonal sub-system, rather than consider the detailed interactions present in the hormonal system as a whole. Having a better understanding of the interactions between various parts of these hormonal sub-systems may ultimately lead to better interpretation of diagnostic tests and also help with the development therapeutics that allow for individualized treatment plans. The aim of this paper is to describe each hormonal element of PCOS in detail, then unify these descriptions into a single codified ‘wiring’ diagram. The unifying diagram is presented at the end of this paper, but a simplified version is shown in **Figure 1**. This simplified version will be used as a basis to construct a more complex representation of the hormonal pathways affected by PCOS upon.

Original research articles were located through topic searches for the relationships between the hormones or biological regions known to be altered in PCOS (**Figure 1**). Clinical research involving humans was prioritized over pre-clinical research using animal models, with animal studies serving as support for human based evidence or suggestions of mechanisms that may underpin the evidence identified in humans. An exhaustive search was not possible due to the volume of research and complexity of the subject. However, effort was made to explore each primary facet of PCOS with evidence from several sources. The ‘wiring’ diagram outcome of this review is intended to capture the major endocrine dysfunctions in PCOS. Specific mechanistic hypotheses are noted where relevant but full coverage of endocrine mechanisms is outside the scope of this review.

## 2 Reproductive hormone changes/dysregulated HPG axis

### 2.1 Hyperandrogenism

**Figure 2:**
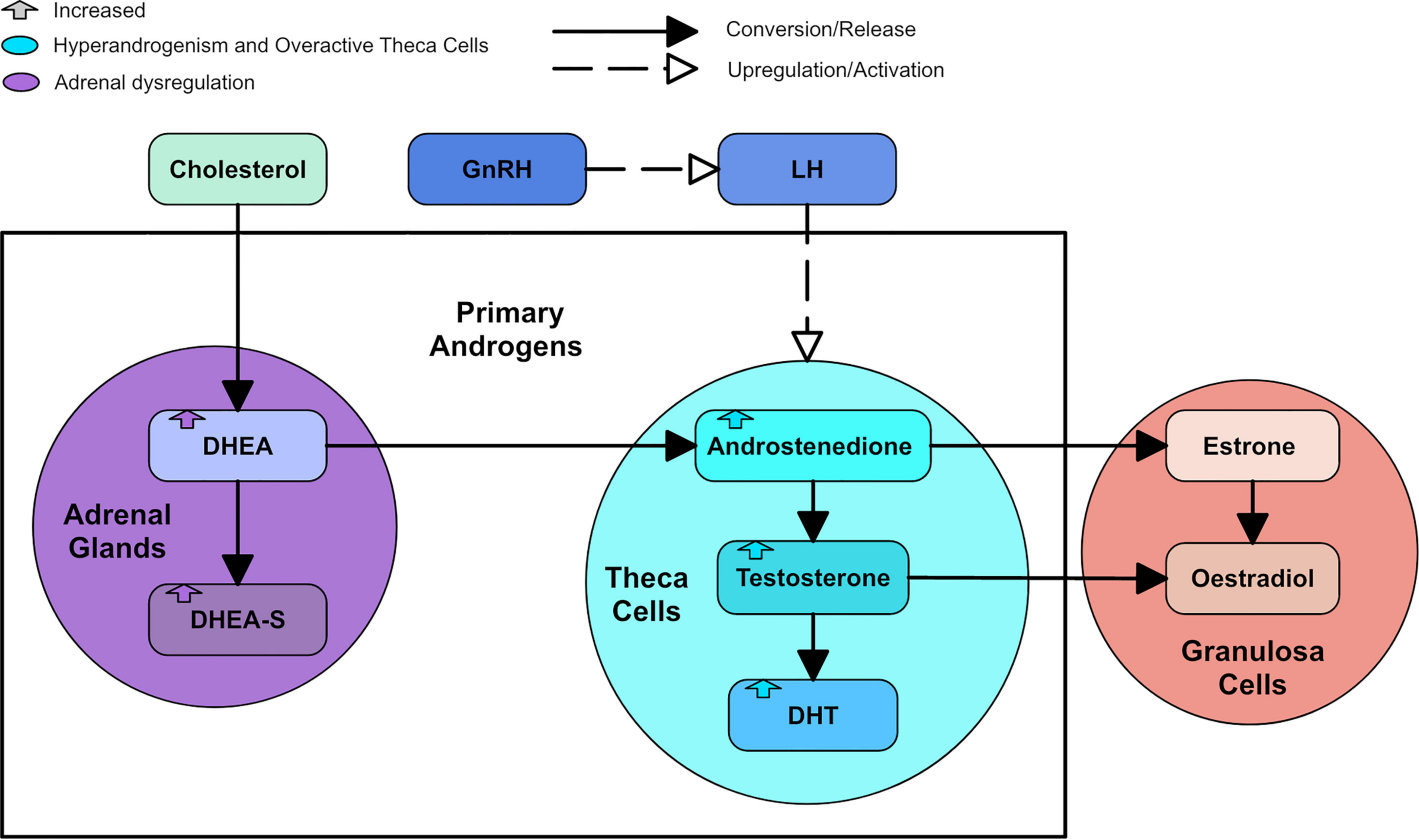
A diagram of the primary androgens, with colored arrows to represent how the androgens are changed by types of dysregulation.

Hyperandrogenism is a key component of PCOS. Approximately 60-80% of women with PCOS present with biochemical evidence of hyperandrogenism using androgen plasma levels and 60% present with clinical evidence of hyperandrogenism such as hirsutism, acne and androgenic alopecia (15, 16). The androgens in women considered to be present in the highest concentrations, in order of most to least concentrated, are dehydroepiandrosterone-sulphate (DHEA-S), dehydroepiandrosterone (DHEA), androstenedione (A4), testosterone and dihydrotestosterone (DHT) (17). DHEA is considered a “weak” androgen that converts to the more potent testosterone and DHT (17). DHEA can be sulphated into DHEA-S, another “weak” androgen with a longer half-life (17). Androgen production in the ovaries is regulated by upstream signals from the hypothalamic region of the brain. Neurons in the hypothalamus secrete pulsatile gonadotropin releasing hormone (GnRH) into the portal vasculature of the pituitary gland (18). GnRH is responsible for stimulating luteinizing hormone (LH) and follicle stimulating hormone (FSH) production and release (17). LH secretion mirrors the pulsatile release of GnRH and stimulates ovarian theca cells to synthesize A4 and testosterone from cholesterol (17). In the ovarian granulosa cell, A4 and testosterone are converted to estrogens through aromatization (17). DHT cannot be converted to estrogen, as it is said to be non-aromatizable, and is the most potent androgen isoform (17). In addition to the ovaries, androgens are synthesized in the adrenal glands, which mainly produce DHEA and A4 (17). About 10-25% of testosterone is produced in the adrenal cortex but this is regarded by some as a negligible contribution to the total concentration of testosterone in a woman (19). **Figure 2** shows a diagram of the primary androgens and their significant relationships.

Androgen excess (hyperandrogenism) is a key feature in PCOS and detrimentally affects ovarian function. Androgens are thought to stimulate pre-antral and small antral follicle growth through the androgen receptor (20). In women with PCOS, the androgen receptor may experience increased activity in the hypothalamus, ovary, skeletal muscle or adipose cells (21). High androgens in PCOS at least partially contribute to an increase in GnRH/LH pulse frequency (1, 22) and vice versa, generating a cycle of hormonal dysregulation (23). Functional ovarian hyperandrogenism can be directly or indirectly identified in the vast majority of PCOS patients (1). Functional adrenal hyperandrogenism is also present in a portion of PCOS patients, with a small percentage of this group not presenting with functional ovarian hyperandrogenism (1). A minor percentage of mostly obese PCOS patients present with neither functional ovarian nor functional adrenal hyperandrogenism. This small subgroup may present an endocrine state that appears to be a phenotype of PCOS but has an etiology related to obesity (excess adipose tissue) (1, 24).

Hyperandrogenism may also influence endometrial function (10). People with PCOS have an altered endometrium (25). This altered endometrium, along with other abnormalities commonly associated with PCOS, may lead to endometrial dysfunction, which can lead to pregnancy complications (25). Reduced reproductive potential in PCOS is often associated with anovulation and oligo-ovulation, but endometrial dysfunction may also contribute (26).

PCOS theca cells appear to have a gene expression profile that is distinct from normal theca cells (27). There is strong evidence that PCOS theca cells have an intrinsic abnormality that causes an overexpression of most steroidogenic enzymes, including LH receptors (1, 28–30). This overexpression leads to the increased androgen production that is ubiquitous with PCOS theca cells (31). Additionally, this increased androgen production appears independent to ovulation status (32).

### 2.2 Follicles and PCOM

A follicle in the ovary is composed of the oocyte, granulosa cells, and theca cells (33). Follicles start as primordial follicles which contain an oocyte surrounded by a single layer of granulosa cells and a basal lamina (34). Hyperandrogenism may have a negative effect on oocyte development and quality through the increase of reactive oxygen species levels (26). However, oocyte competence and quality appear equivalent in people with and without PCOS, so whether the oocyte contributes to decreased reproductive potential in PCOS is unclear (26). There are no theca cells until 2-3 layers of granulosa cells are acquired (34). There are large antral (later stage) and small pre-antral (earlier stage) follicles in the ovary. The density of pre-antral and small antral follicles in a person with Polycystic ovarian morphology (PCOM), one of the three diagnostic criteria of PCOS, has been shown to be six times greater than in a normal ovary (35).

**Figure 3:**
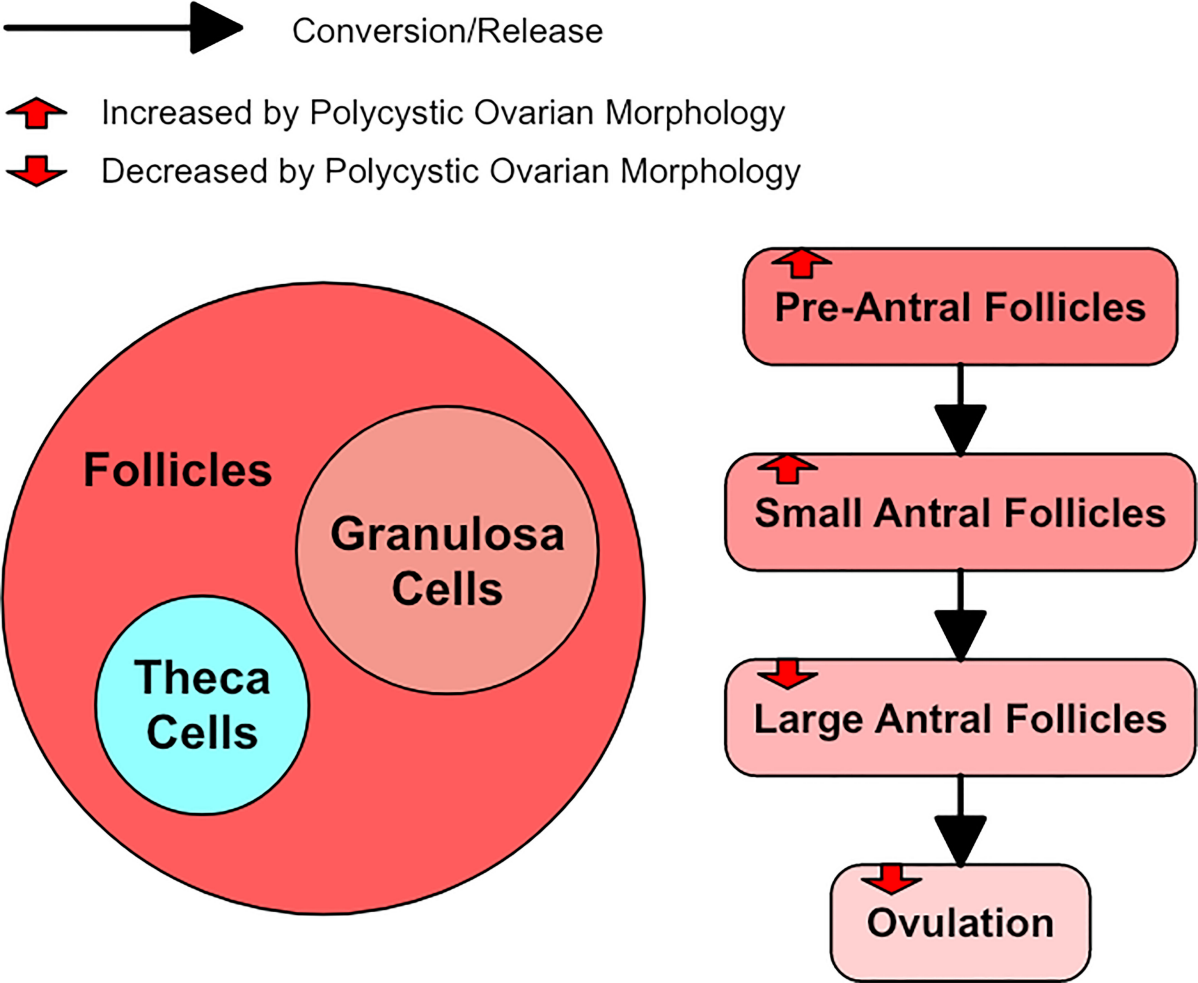
A diagram of follicle growth, with red arrows to represent how polycystic ovarian morphology alters it.

While PCOM may occur in PCOS, PCOM can also occur in women who do not fully meet the diagnostic criteria for PCOS. In such cases, PCOM generally has a granulosa cell abnormality that is similar to PCOS but not as severe (36). Normal ovarian morphology and PCOS seem to exist on a spectrum with PCOM sitting between them (1). PCOM groups have been shown to have LH levels similar to non-PCOS and non-PCOM controls, but their anti-Mullerian hormone (AMH) levels were between that of controls and PCOS patients (36). Approximately half the women with PCOM have subclinical evidence of PCOS-related dysregulation whereas the other half have no apparent relation to PCOS, but most women with PCOM appear to be ovulatory (1). **Figure 3** shows a simple diagram of follicle growth.

### 2.3 Increased LH synthesis over FSH synthesis

Serum LH concentrations have been found to be significantly increased in women with PCOS (37, 38). In contrast, FSH concentrations have been significantly lower in women with PCOS (39). Consequently, elevated LH/FSH ratios are generally reported (37, 38, 40). One study of 192 women showed that LH and FSH appear lower in women with PCOS who are also overweight or obese when compared to women with PCOS that were in the healthy BMI range (41). Among women in the healthy BMI range, those with PCOS had significantly higher FSH concentrations than controls (41). However, the women in the overweight or obese BMI categories had significantly lower FSH concentrations than controls (41).

Increased LH secretion is thought to have several consequences. LH stimulation of thecal cells in the ovary drives the synthesis of testosterone. Thus, the androgen synthesis in ovarian thecal cells is promoted, and can lead to hyperandrogenism. Elevated serum LH concentration is closely associated with a reduced chance of conception and an increased risk of miscarriage (42). However, it is unclear how substantially excessive LH in PCOS contributes to ovarian dysfunction (23).

**Figure 4:**
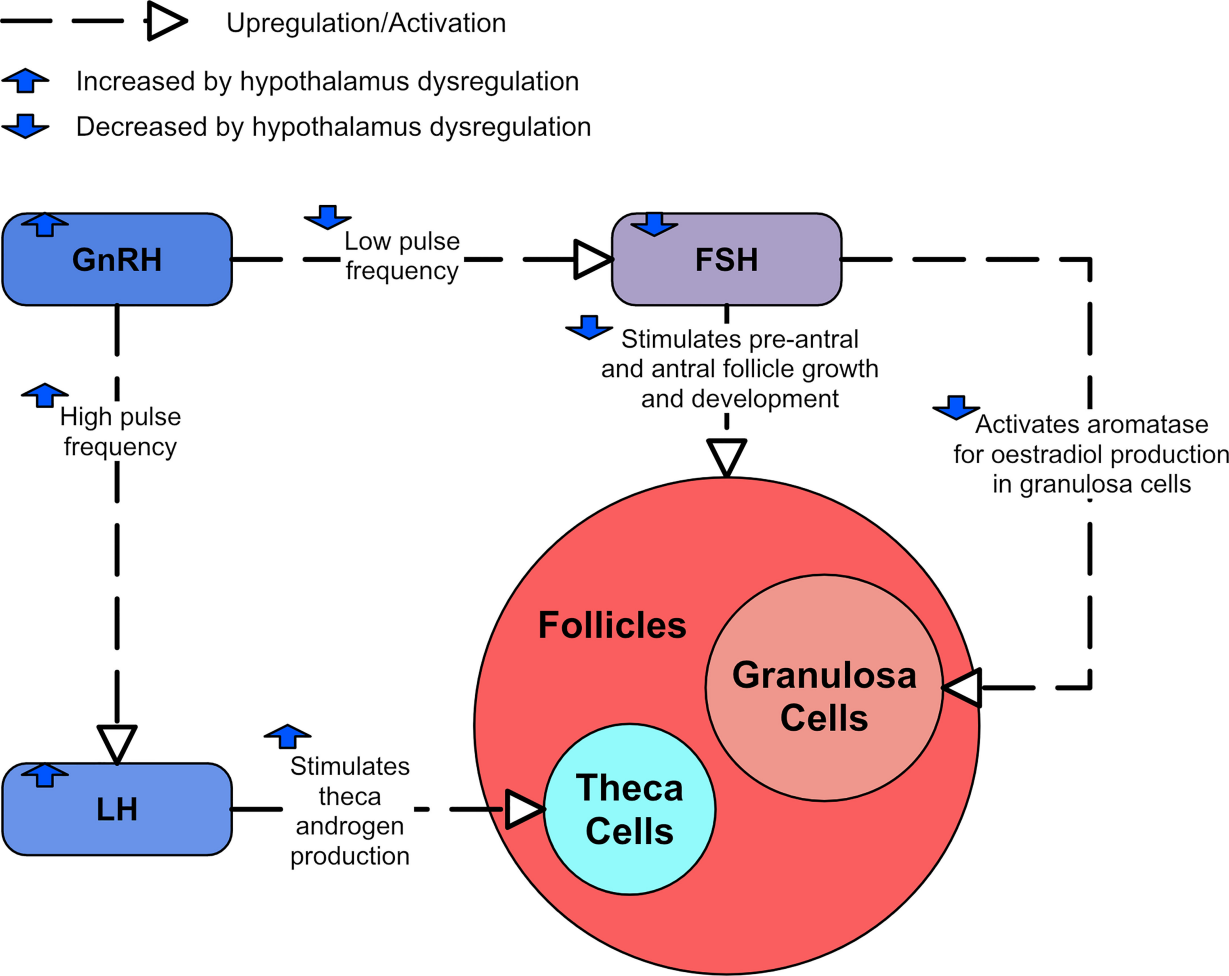
A diagram showing the effects of LH and FSH dysregulation in PCOS.

The A4 and testosterone formed by the thecal cells in response to LH stimulation is converted in the granulosa cell to estradiol, but the activation of the enzyme aromatase necessary for this estradiol synthesis is dependent on FSH (43). FSH also promotes pre-antral follicle growth in synergy with theca cell-derived androgen. In PCOS, both these functions become dysregulated (44). Serum concentration and follicular fluid concentrations of FSH are generally lower in PCOS, with some studies reporting significantly decreased levels (45), and others reporting lower levels that are still within normal limits (46). However, these lower FSH levels do not seem to be enough to account for the disturbance of antral follicle growth and estradiol synthesis (47). **Figure 4** shows the consequences of dysregulated LH and FSH.

### 2.4 Increased GnRH pulse frequency

LH release is a result of pulsatile GnRH transfer from the hypothalamus to the pituitary (48). GnRH neurons in the hypothalamus are controlled by gonadal steroid feedback and release patterned pulsatile GnRH peptide to maintain pituitary function. A high pulse frequency of GnRH secretion favors release of LH while a low frequency of GnRH secretion favors greater FSH release. Thus, increased GnRH pulse frequency promotes LH synthesis over FSH synthesis (38, 49). Increasing levels of estradiol produced during the majority of the follicular phase cause a switch to positive feedback, triggering ovulation with an increase in LH (50). Ovulation triggers the increase in progesterone during the luteal phase, and low levels of progesterone are used as a clinical indicator that ovulation has not occurred (51). During the luteal phase, progesterone and estradiol produced by the corpus luteum signal the lowering of the GnRH pulses *via* negative feedback (50, 52) and by increased progesterone levels in the presence of estradiol (53). Evidence in the ewe suggests that estradiol alone inhibits GnRH and LH pulse amplitude, whereas progesterone alone inhibits GnRH and LH pulse frequency (54). Androgens have also been shown to contribute to increasing GnRH pulse frequency (55).

In PCOS, the sensitivity of the GnRH pulse generator to progesterone suppression is impaired (56, 57). Thus, ovarian steroid negative feedback that tightly regulates the Hypothalamic-Pituitary-Gonadal (HPG) axis is impaired (58), leading to hyperactive GnRH/LH secretion and disruptions to the neuroendocrine regulation of the reproductive system. Mechanistic studies in rodents demonstrate that DHT impairs the ability of progesterone to reduce the firing frequency of GnRH neurons from the HPG axis (59).

**Figure 5:**
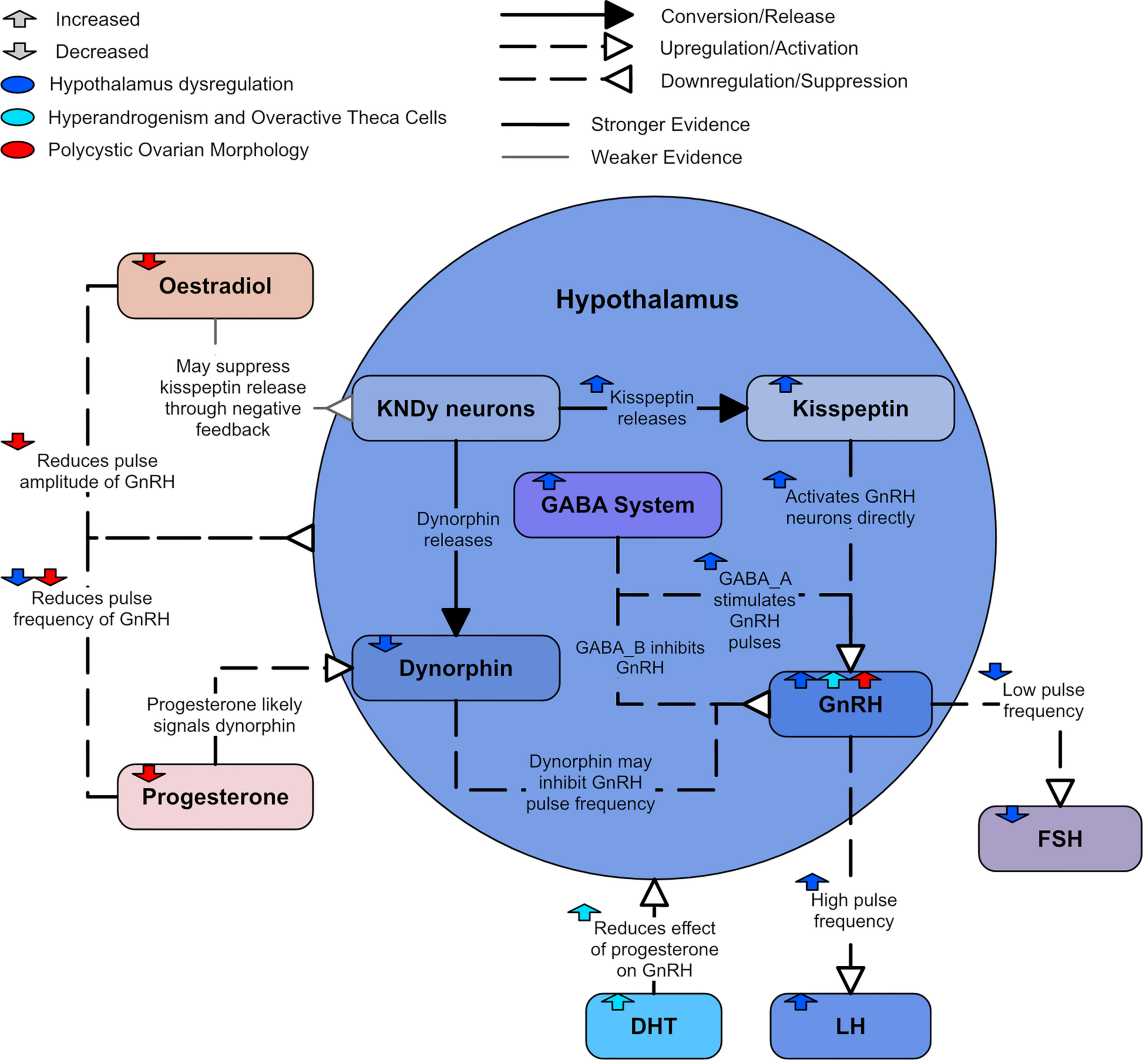
A diagram of hypothalamic dysregulation in PCOS.

The mechanisms underpinning increased GnRH activity are not completely understood but have been explored in animal models. **Figure 5** displays a summary of the likely hypothalamic dysregulation. One potential contributor is the GABAergic network. γ-aminobutyric acid (GABA), the major inhibitory neurotransmitter in the brain, binds to two primary receptors: the GABA_A_ receptor, and the GABA_B_ receptor (60). While GABAergic signaling is predominantly inhibitory in the adult brain, GABA has a predominantly stimulatory effect on GnRH neurons through the GABA_A_ receptor (50, 60), but an inhibitory effect through the GABA_B_ receptor can also be observed (60). Increased GABA signaling to GnRH neurons has been identified in preclinical models of PCOS (61, 62) and cerebrospinal fluid GABA levels are higher in women with PCOS (63). There is a strong suggestion that this increased GABA signaling plays a role in driving the hyperactive GnRH release associated with PCOS as the GABAergic system is also thought to play a role in progesterone negative feedback (59). Hyperandrogenic mouse models of PCOS were shown to have significantly less progesterone receptors in a population of GABA neurons and exhibited impaired progesterone negative feedback (64, 65). This decrease in progesterone receptors likely contributes to the impaired progesterone inhibition of GnRH secretion found in some women with PCOS (66). Flutamide, an androgen receptor blocker, restores normal GABA innervation and neurotransmission in hyperandrogenic mice models (61, 62) and sensitivity to progesterone in women with PCOS (67). Mice models have also been used to demonstrate that DHT may increase the pulse frequency *via* the GABAergic system (55).

Kisspeptin, neurokinin B, and dynorphin are three distinct neuropeptides that are found co-localize in a single subpopulation in the hypothalamus of several mammalian species. They are referred to as kisspeptin-/neurokinin B-/dynorphin-expressing or KNDy neurons. There is strong evidence that KNDy neurons have an important role in mediating the negative feedback of GnRH secretion by ovarian steroids (22, 68). It is possible that estradiol inhibits GnRH pulse amplitude by suppressing kisspeptin release from KNDy neurons (69). The kisspeptin neuropeptides that release from KNDy neurons have been identified as a primary GnRH pulse generator (50).

There is evidence that kisspeptin may contribute to GnRH neuron hyperactivity (22). Kisspeptin potently and directly activates GnRH neurons and drives GnRH/LH secretion (70, 71). Mice models have suggested that the presence of estradiol leads to kisspeptin indirectly activating GnRH neurons as well (70). It has been found that women with PCOS had increased kisspeptin levels, suggesting that kisspeptins play an important role in regulating LH levels (72, 73). It is unclear if this elevated kisspeptin is related to increased kisspeptin signaling in the brain. Animal models suggest not all PCOS phenotypes present with high kisspeptin levels (73). However, the models imply kisspeptin is elevated in PCOS with higher LH levels and normal body weight (73).

There is also considerable evidence that dynorphin is an important mediator of the inhibitory feedback control of progesterone on GnRH secretion (54). Evidence in the ewe shows a very high percentage of dynorphin neurons contain progesterone receptors; as such, dynorphin is likely to inhibit GnRH pulse frequency (74). Dynorphin expression has been found to be reduced in ewes prenatally exposed with androgen (75), which is in agreement with the theory that PCOS may be caused by prenatal exposure to excess androgens (76, 77).

### 2.5 Increased AMH

The formation of pre-antral and small antral follicles is thought to be accelerated by the hyperandrogenism present in PCOS (78). Granulosa cells are considered the only source of AMH in the ovary (79). Mice models have shown that AMH is released throughout the development of a primordial follicle into a small antral follicle but AMH release wanes as the follicle develops into a pre-ovulatory stage (78). As there are often more pre-antral and small follicles in PCOS ovaries, more AMH is generally produced than in normal ovaries (47). Furthermore, each individual follicle in PCOS has been shown to produce more AMH than normal (47). However, this increased AMH may not be solely due to PCOM. In particular, serum AMH has been positively correlated to high androgen levels, and women with high androgens and PCOM seemed to have the highest AMH levels of women with PCOS (79). Therefore, high androgens may also contribute to increased AMH levels.

FSH promotes the development of small antral follicles through to an ovulatory stage (47). AMH has been shown to both inhibit FSH-induced aromatase activity and counteract FSH growth-promoting effects on granulosa cells (44, 80), consequently deterring estradiol production (79). In PCOS, antral follicle growth can be disturbed by high AMH levels inhibiting FSH effects (80). Thus, FSH-stimulated pre-antral follicle growth is attenuated (81). This suggests that increased AMH levels likely play a role in the causation of anovulation in PCOS (47). AMH has also been shown to contribute to GnRH hyperactivity in animal models, as it can directly activate GnRH neurons (82). As there is a negative correlation between estradiol and AMH in small antral follicles, AMH is thought to be down-regulated by FSH, potentially *via* estradiol synthesis (83).

**Figure 6:**
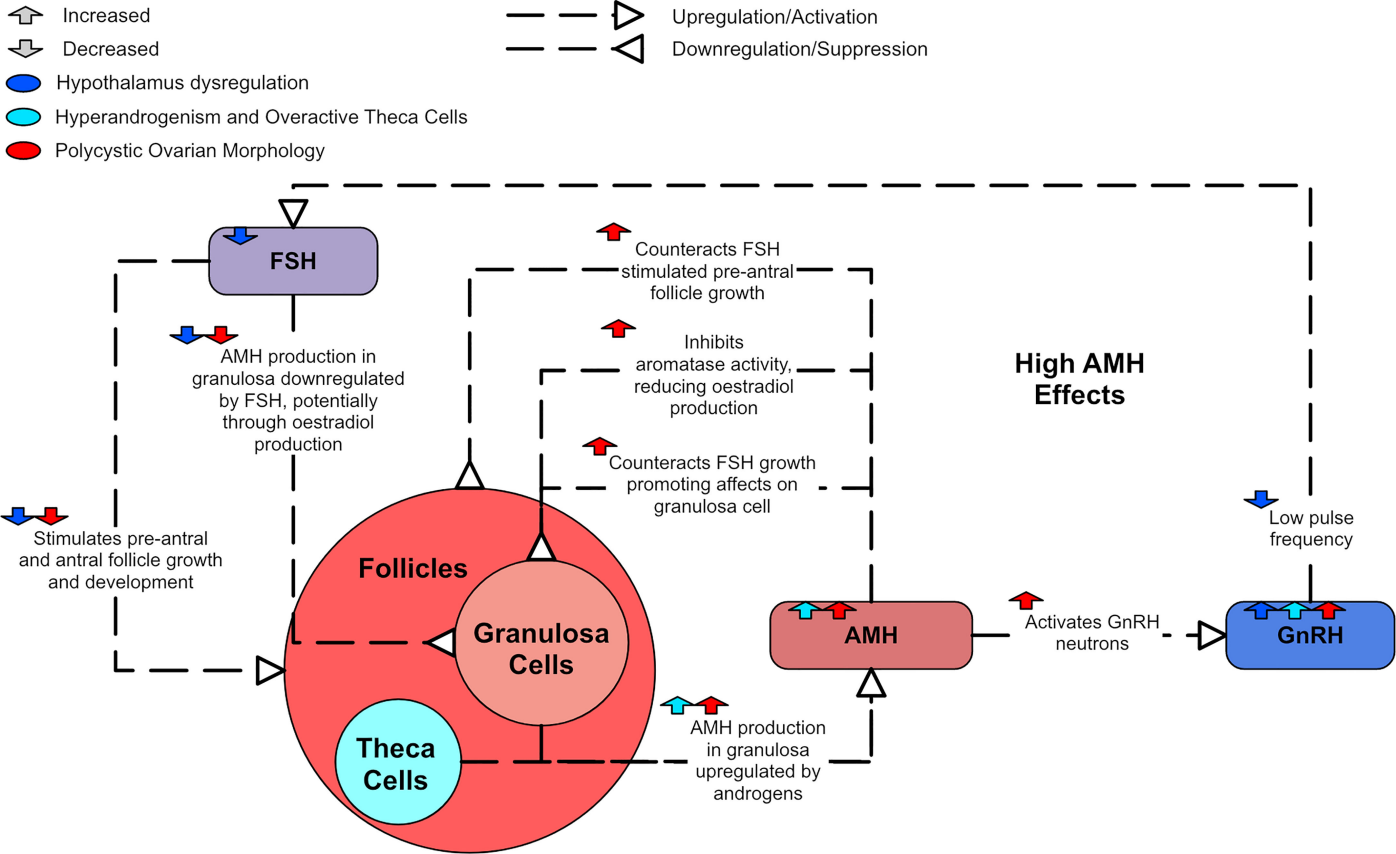
A diagram of the effects of AMH in PCOS.

Serum AMH concentrations are higher in PCOS patients and there is evidence to suggest that more severe PCOS phenotypes display higher AMH levels (46, 84, 85). One study of 104 women found that PCOS patients with amenorrhea had higher AMH serum concentrations than those with oligomenorrhoea (86). Similarly, another study of 215 women found that oligo/anovulatory women with PCOS had higher AMH levels than ovulatory women with PCOM (36). **Figure 6** shows how high AMH relates to the dysregulation in PCOS.

### 2.6 Increased DHEA and DHEA-S

In humans, almost all A4 is produced from DHEA (19), which is produced in the adrenal gland. It is commonly believed that DHEA and DHEA-S can freely convert between each other, with DHEA-S acting as a reservoir for DHEA due to its long half-life (17). However, this belief has been challenged by a study finding no evidence of a rise in DHEA-S upon DHEA administration in men (87). The study also noted that although pregnant women have been shown to convert DHEA-S to DHEA, they were unable to find direct evidence of continuous conversion between DHEA and DHEA-S in women in the literature (87).

Since DHEA-S is almost solely produced by the adrenal cortices, increased DHEA-S is often used as an indicator for overactive adrenal cortex production of androgens. Approximately 50-60% of women with PCOS exhibit adrenal originating androgen excess by increased DHEA-S (88). Currently, it is thought the ovary has a limited to negligible effect on the adrenocortical function (89). DHEA-S has been found to positively correlate with total testosterone, A4, and free androgen index (FAI) (90), as well as 17-hydroxyprogesterone (91). DHEA-S has also been found to decline with age (89, 92).

Insulin and insulin resistance will be discussed directly in section 3. However, there are complex links between DHEA-S and insulin that will be discussed in this section. Some studies negatively correlate DHEA-S and homeostasis model assessment-estimated insulin resistance (HOMA-IR) (89, 91). Other studies report no association (90), or a positive correlation between higher adrenal precursor androgen levels and insulin resistance (89). However, the HOMA-IR metric is imprecise and can fail to identify insulin resistance in woman with PCOS when compared to the gold standard of insulin resistance detection (93). There may also be ethnic differences, with one study finding that those they classified as white patients displayed a negative correlation between DHEA-S and HOMA-IR while those they classified as black patients did not (89). However, the number of patients in the ‘black’ cohort was too low to allow strong conclusions.

**Figure 7:**
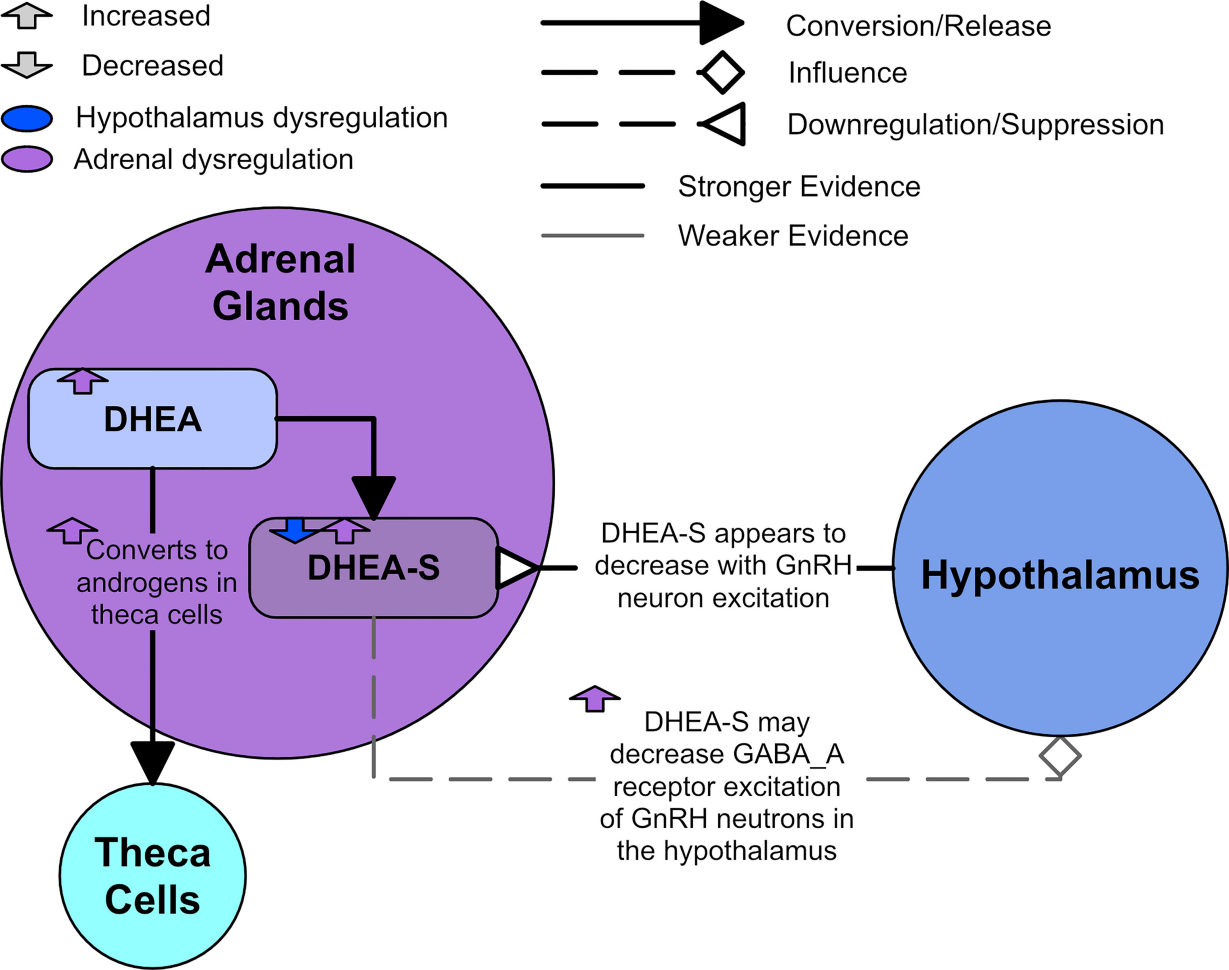
Adrenal dysregulation in PCOS.

There is evidence to suggest communication between the adrenals and the hypothalamus. DHEA-S levels have been observed to decrease after administration of a GnRH antagonist in women with PCOS and high DHEA-S (94). Sullivan and Moenter reported that DHEA-S decreases GnRH neuron excitation through modulating the GABA_A_ receptors in mice (95). It was also found that asymptomatic ovulatory women with PCOS had the highest DHEA-S levels when compared to other women with PCOS and those without PCOS (1). **Figure 7** shows a diagram of the effects of an overactive adrenal cortex.

## 3 Metabolic hormone changes

PCOS is strongly associated with insulin resistance – around 75% of women diagnosed with PCOS also have impaired insulin sensitivity (93). Furthermore, lean women with PCOS have equivalent peripheral insulin resistance to obese women with PCOS (96). Thus, women with PCOS are vulnerable to developing metabolic syndrome and its associated dysfunction, which include hyperglycemia, central obesity, hypertension, and dyslipidemia. Metabolic syndrome is typically caused by interactions between insulin resistance and obesity. PCOS-linked insulin resistance is more severe in hyperandrogenic PCOS than non-hyperandrogenic PCOS (97). The pathogenesis of metabolic dysfunction in women with PCOS is not fully understood, but there is suggestion that hyperandrogenism influences the metabolic facets of PCOS (98, 99).

### 3.1 Insulin resistance and hyperinsulinemia

Insulin resistance induces hyperinsulinemia that can exacerbate PCOS dysfunction. Insulin and testosterone levels appear highly correlated and hyperinsulinemia has been suggested as the primary cause of increased testosterone (100). Insulin resistance also significantly increases the risk of T2DM (4, 5). Furthermore, obstructive sleep apnea is a PCOS comorbidity and is known to exacerbate insulin resistance (1). As with typical insulin resistance, PCOS-related insulin resistance is characterized by reduced sensitivity and responsiveness to insulin-mediated glucose utilization primarily in skeletal muscle and adipose tissue (101). Of interest, insulin sensitivity is not diminished in the ovaries, pituitary gland, or the adrenal gland in women with PCOS.

**Figure 8:**
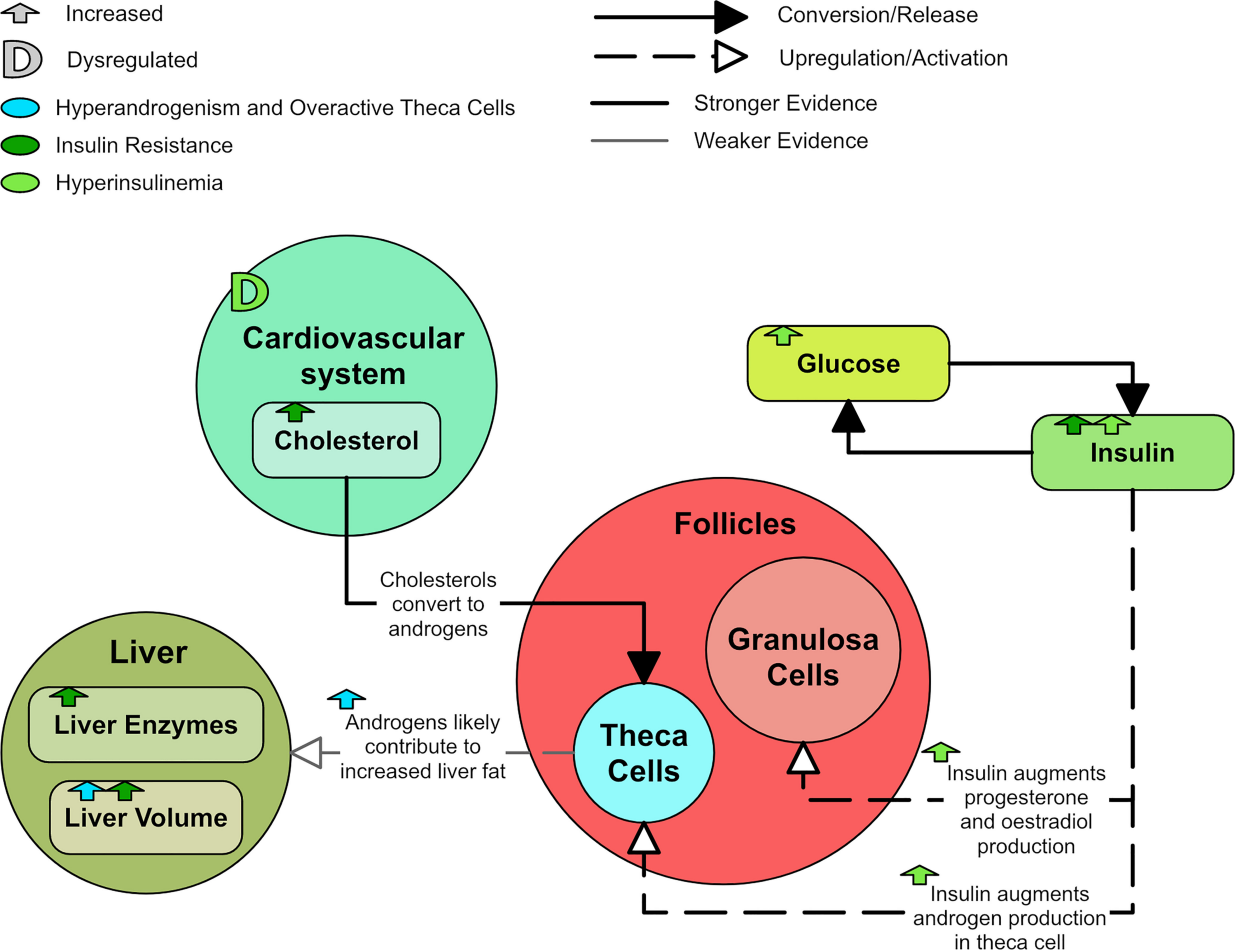
A diagram of the effects of insulin resistance and hyperinsulinemia in PCOS.

Insulin is thought to augment LH-stimulated testosterone production through activation of its receptor (102, 103). Rat studies have shown hyperinsulinemia upregulates LH-binding sites, thus augmenting LH-induced testosterone production in theca cells (34, 104, 105). Insulin in rats also augments the GnRH-stimulated production of LH, which has been shown to be glucose dependent (103). Insulin seems to augment the production of estradiol and progesterone in human granulosa cells through its own receptor (106). Therefore, elevated insulin can promote and exacerbate hyperandrogenism and PCOS symptoms (**Figure 8**).

A relationship between insulin resistance, hyperinsulinemia and hypertension has been observed, leading to the postulation that hyperinsulinemia contributes to cardiovascular disease through direct mechanisms such as increasing sympathetic activity (107). Hyperinsulinemia may also contribute to cardiovascular disease by inducing abnormalities in endothelial function and vascular reactivity (108). In a study of over 2,000 PCOS patients, total cholesterol, triglycerides and low-density lipoprotein (LDL) cholesterol were significantly higher in PCOS patients than in controls even after correcting for body mass index (BMI) (39).

PCOS and its comorbidities are known to affect the liver. Insulin resistance (109) and PCOS (14) are compounding risk factors for NAFLD. In particular, PCOS doubles the risk factor for NAFLD in women (14). The liver enzymes aspartate transaminase (AST) and alanine transaminase (ALT) are significantly higher in PCOS groups (39) and are known markers of liver disease. Androgen excess has also been shown to contribute to the occurrence of NAFLD in PCOS rat models (110). It has been demonstrated that hyperandrogenic women with PCOS have increased liver fat compared to non-hyperandrogenic women with PCOS and non-PCOS controls, even after correction for BMI, adipose tissue volume, and HOMA-IR (98, 109). There is suggestion that an androgen-dependent proapoptotic PCOS environment contributes to NAFLD (111).

### 3.2 Decreased IGFBP-I and increased IGF-I bioactivity

Insulin-like growth factor I (IGF-I) is produced in theca cells (33) and has been shown to stimulate testosterone production (112), likely by increasing LH binding affinity with theca cells (104). IGF-I may also augment FSH stimulated production of estradiol (113). Insulin amplifies these effects as it suppresses insulin-like growth factor binding protein I (IGFBP-I) production (114). This suppression of IGFBP-I enhances IGF-I bioactivity (114). LH is also thought to promote ovarian secretion of IGF-I (104) and DHEA-S has been found to positively correlate with IGF-I (91).

**Figure 9:**
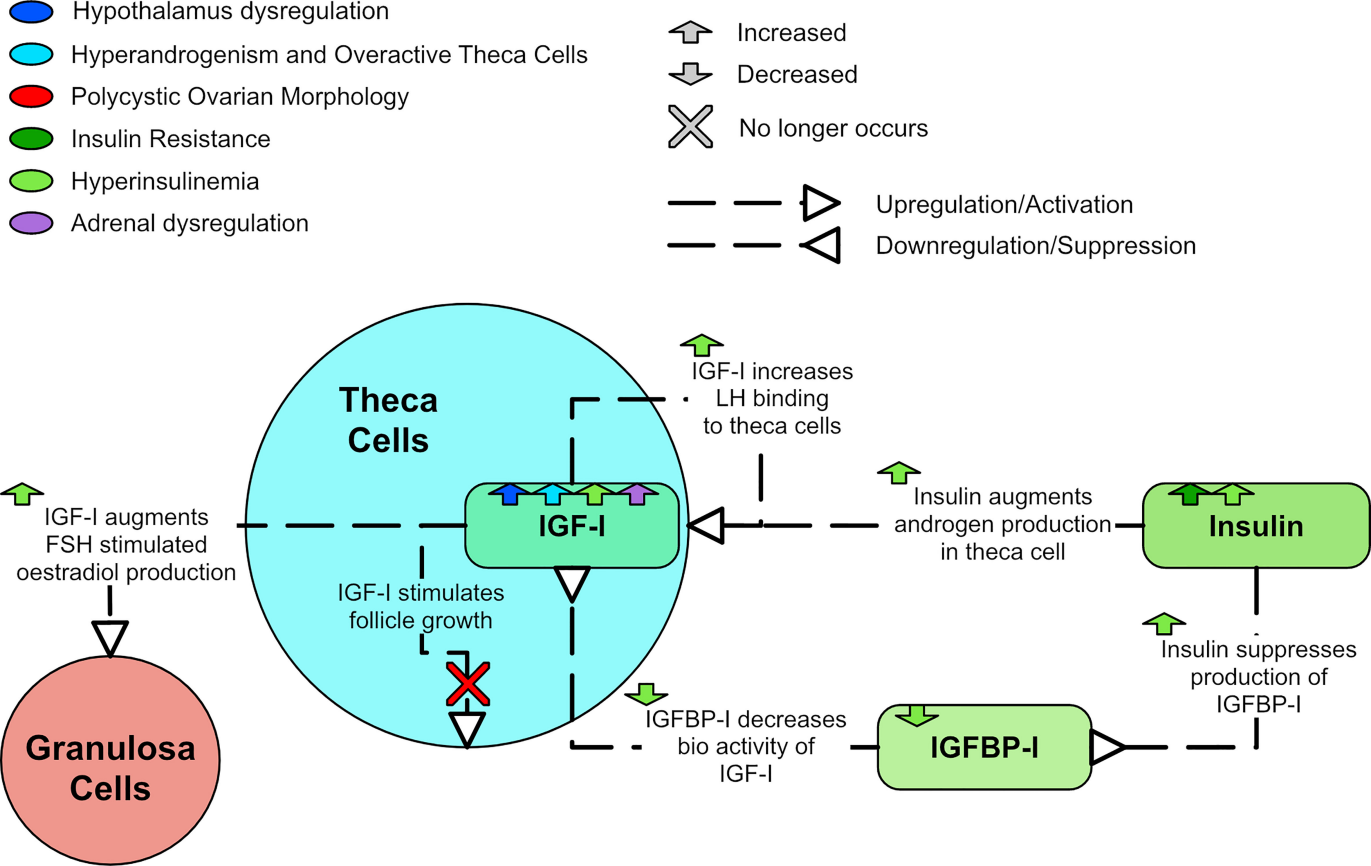
A diagram of the relationships of IGF-I and IGFBP-I in PCOS.

A meta-analysis found that women with PCOS appear to have lower levels of IGFBP-I than controls (115). However, adjustments for BMI suggested that decreased IGFBP-I may be the result of obesity and not have a role in the pathogenesis of PCOS (115). Thus, the relationship between IGF-I, IGFBP-I and PCOS remains unclear. IGF-I, IGF-II, and insulin can each augment LH-induced A4 production in the theca cell. Thus, it is possible for insulin resistance to have a role in hyperandrogenism, even in the absence of hyperinsulinemia (116). IGF-I has been shown *in vitro* to stimulate follicle growth in normal human ovaries but not polycystic ovaries (117). **Figure 9** shows the dysregulation of IGF-I and IGFBP-I.

### 3.3 Decreased SHBG

**Figure 10:**
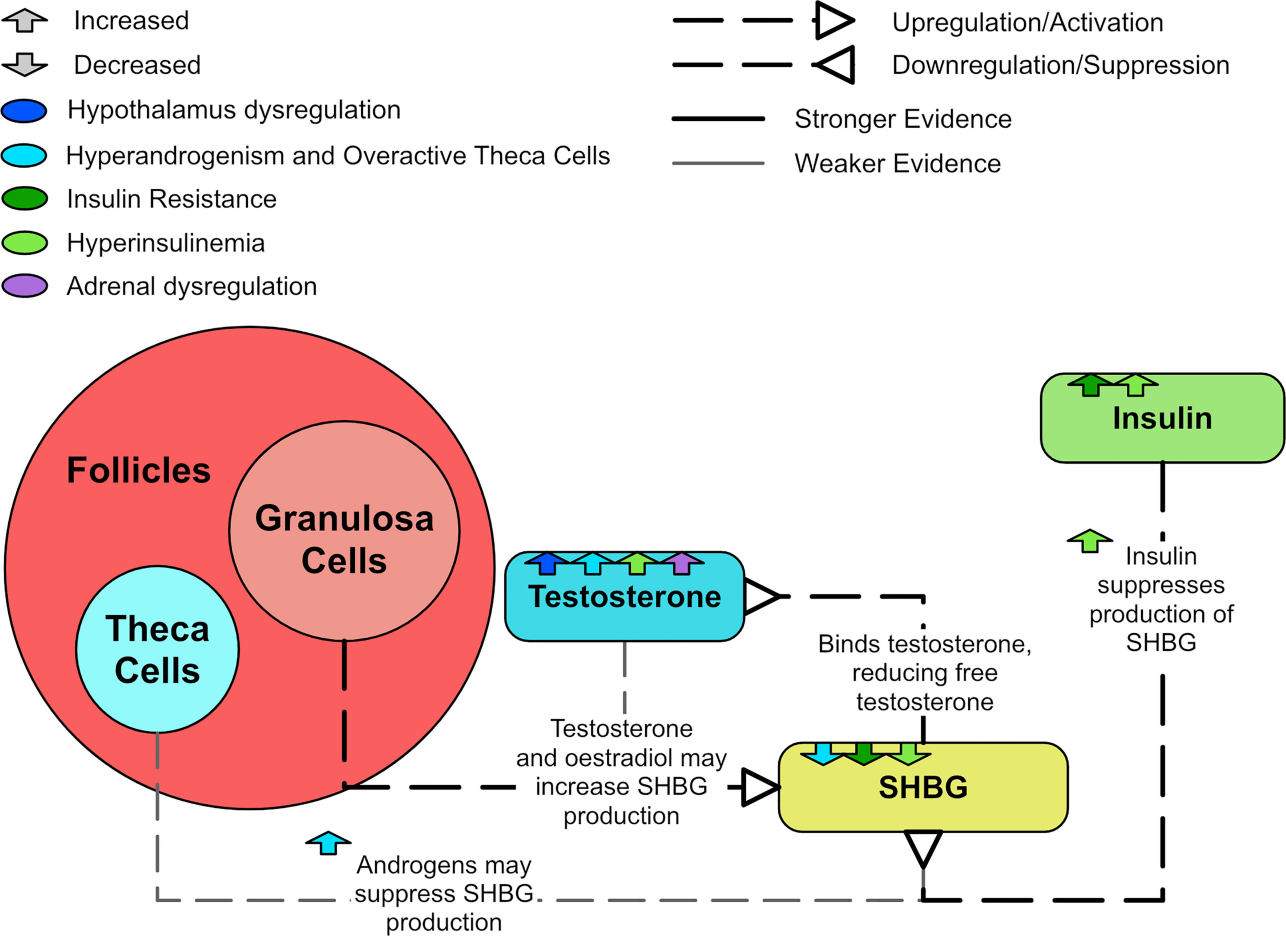
A diagram explaining SHBG and PCOS.

Low concentrations of sex hormone binding globulin (SHBG) are prevalent in T2DM, impaired glucose tolerance, insulin resistance, and obesity (118). Most circulating testosterone and estrogen can bind to SHBG, preventing the hormones from entering cells and binding to their receptors (17). Insulin and insulin resistance suppresses SHBG production (102, 119), resulting in increased circulating free testosterone levels and enhanced hyperandrogenism. SHBG levels have been shown to be raised by estrogen and possibly suppressed by androgens (120). However, testosterone has also been shown *in vitro* to increase SHBG levels to the same degree that estradiol does (119). A meta-analysis determined that SHBG levels are decreased in women with PCOS and appear to increase with treatments that improve their endo-metabolic profile (118). The meta-analysis also suggested that women with PCOS and lower SHBG levels were more likely to have hyperandrogenism, metabolic issues and infertility (118). SHBG levels are especially lower in those who also possess a high BMI (121–123). **Figure 10** shows how SHBG is dysregulated in PCOS.

### 3.4 Thyroid and autoimmune disorders

**Figure 11:**
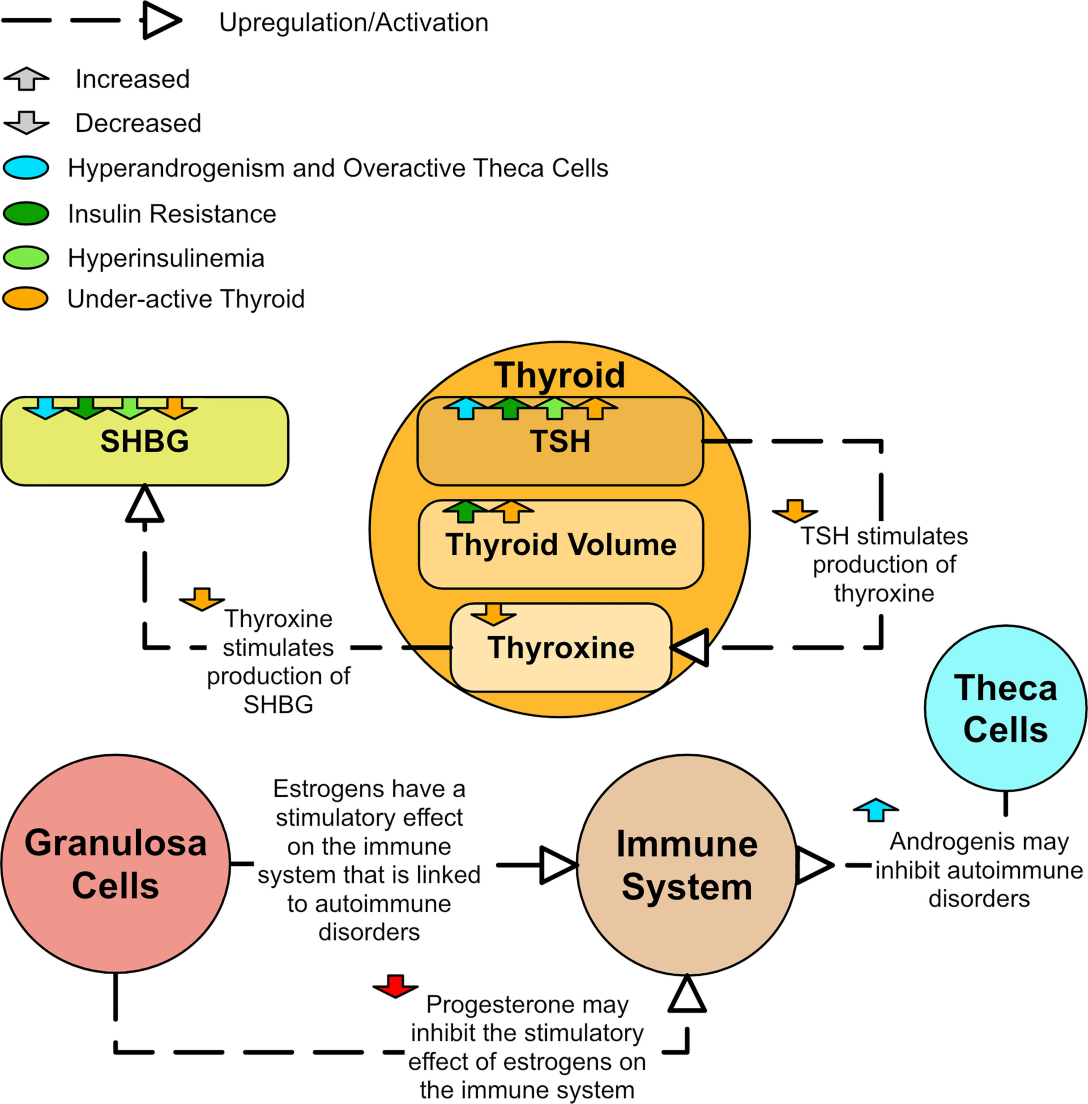
A diagram of how the thyroid and immune system could be dysregulated in PCOS.

Thyroid disorders, especially the autoimmune disorder Hashimoto’s thyroiditis (HT), are observed significantly more frequently in PCOS patients than in the general population (12). Patients with both PCOS and HT have more severe metabolic symptoms than patients with either condition in isolation (12). A study of 125 women found that women with PCOS had increased thyroid volume compared to controls and the volume was highest in the insulin resistant PCOS group (124). In a study of 800 women, levels of thyroid stimulating hormone (TSH) were found to be higher in women with PCOS and appeared to be associated with hyperandrogenism (125). High TSH is a marker for an underactive thyroid and underproduction of the thyroid hormone, thyroxine. In a study of 103 women with PCOS, associations were found between high TSH values and high BMI, increased fasting insulin, high HOMA-IR indices, high testosterone, high FAI, and low SHBG (126). A study of 164 women found that those with PCOS and HT had lower SHBG than those with PCOS alone and normally functioning thyroids (12). This reduced SHBG in HT could occur because thyroxine has been shown to have a stimulatory effect on SHBG *in vivo* (119). **Figure 11** shows the major aspects of dysregulation of the thyroid observed in PCOS.

The link between Hashimoto’s and PCOS is well established, but there also appears to be a link between PCOS and autoimmune disorders in general (127–129). Certain rheumatic diseases, which are autoimmune and inflammatory diseases, are more prevalent in PCOS (129). Some even claim that PCOS could be classed as an autoimmune disorder (128). Excess estrogen has been linked to different autoimmune diseases (128). The stimulatory effect of estrogen on the immune system may be inhibited by progesterone (127). As such, low levels of progesterone could lead to an overstimulated immune system (128) even though estradiol is not necessarily elevated in PCOS. However, high levels of androgens in PCOS appear to have a protective role against development of autoimmune disorders (127). Therefore, it is likely that the association of PCOS with autoimmune disorders differs by phenotype.

### 3.5 Low prolactin and hyperprolactinemia

While prolactin is primarily known for regulating breast development and lactation, it has many other functions and is closely associated with metabolism. Prolactin secretion from the pituitary is stimulated by estradiol and regulated by the hypothalamus (130). Prolactin synthesis can also occur elsewhere in lower quantities, including from adipose tissue and the uterus (131). An *in vivo* study involving rats and humans found that high glucose and inflammation may stimulate the synthesis of prolactin in adipose tissue (132).

Hyperprolactinemia has been shown to induce an insulin resistant state in non-PCOS cohorts (133, 134). Prolactin also appears to inhibit SHBG production (119) and positively correlate with TSH (39). Due to the possibility that hyperprolactinemia may mimic PCOS, it is often one of the exclusion criteria in PCOS diagnosis (135). However, approximately 20% of women with PCOS also have hyperprolactinemia (135, 136). Women with coincident PCOS and hyperprolactinemia appear more insulin resistant than women with PCOS and normal prolactin levels (137). Some believe hyperprolactinemia is not more frequent in women with PCOS and that any association between them is casual (136). Others believe that hyperprolactinemia may be an integral part of PCOS (135) and PCOS with hyperprolactinemia may be a specific phenotype of PCOS.

**Figure 12:**
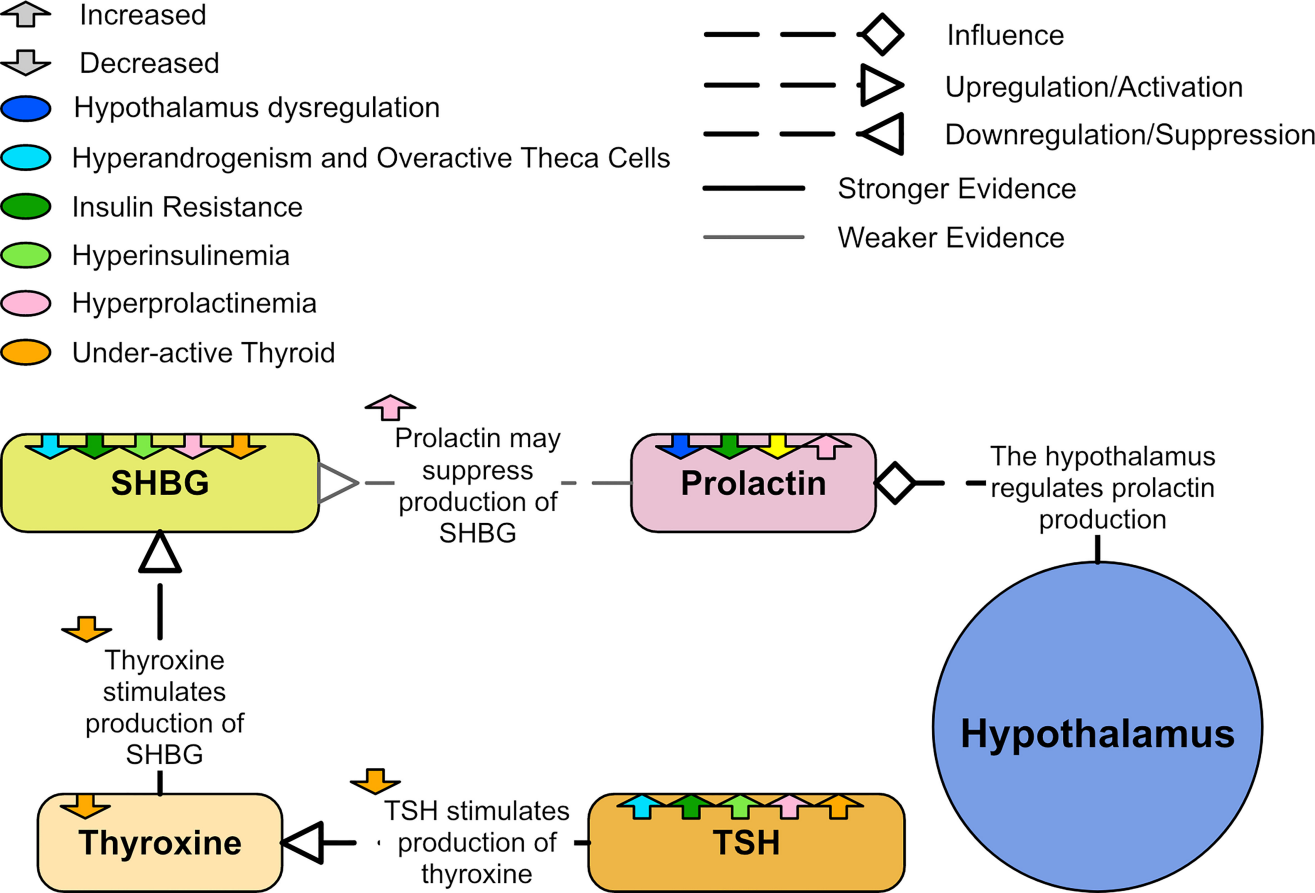
A diagram of showing the relationships to prolactin in PCOS.

While a subset of women with PCOS have high prolactin levels, a study of 2,052 PCOS patients and 9,696 controls found that levels of prolactin are significantly lower in women with PCOS when compared to controls before and after BMI adjustment (39). Due to the heterogeneous nature of PCOS, it is possible that both hyperprolactinemia and low prolactin are associated with different phenotypes of PCOS (**Figure 12**). Low prolactin seems to correlate with high BMI (138) and this effect appeared more significant in women with PCOS (41). In women with PCOS, prolactin appears to negatively correlate with total cholesterol, triglycerides and LDL cholesterol (39).

Low prolactin seems to be an effective marker of a poor metabolic spectrum and high cardiovascular risk (39). Low prolactin levels are associated with metabolic syndrome and T2DM, while higher prolactin levels within the normal range appear to improve insulin sensitivity (139, 140). Prolactin negatively correlates with AST and ALT, suggesting that low prolactin may damage liver cells (39). Prolactin also plays a role in suppressing stress and anxiety (130). There are strong associations between PCOS and poor mental health. One meta-analysis found that women with PCOS are three times more likely to have depressive symptoms and five times more likely to have anxiety symptoms than controls (13).

### 3.6 Adipose tissue dysfunction

Adipose tissue of women with PCOS is characterized by hypertrophic adipocytes and impairments in lipolysis and insulin action (99). The expression and secretion of a wide variety of adipokines implicated in insulin resistance are altered in PCOS (99). In particular, adiponectin, an adipose-specific protein, is downregulated in obesity and lower adiponectin levels are associated with insulin resistance (141–144). Women with PCOS are reported to have lower adiponectin levels compared with BMI-matched controls (141). Women with PCOS also appear to have larger adipocytes than BMI matched controls. These large adipocytes are also more prevalent in people with a genetic predisposition to T2DM (145). Large adipocytes are strongly correlated with insulin resistance (142). Adipose tissue dysregulation and insulin resistance seems to correlate more strongly with enlarged adipocytes rather than obesity itself (145). Adipocyte size is also inversely correlated with adiponectin levels and Glucose transporter type 4 (GLUT-4) expression (145).

GLUT-4 content in adipocyte membranes is independently decreased by obesity and by PCOS (146). Decreased expression of GLUT-4 leads to decreased insulin sensitivity and responsiveness of the adipocyte to insulin (145). Since diminished adipocyte insulin responsiveness in PCOS is associated with decreased GLUT-4 abundance (146), the problem may compound itself, and lead to divergence from healthy glucose homeostasis.

Leptin is another adipokine that can also be produced from granulosa cells (147). Leptin plays a key role in regulating appetite and energy expenditure (99). Leptin also plays a role in reproductive and immune function (99), as well as insulin action and lipid metabolism (148). Leptin may have an inhibitory effect on IGF-I augmentation of FSH-stimulated granulosa cell production of estradiol and LH-stimulated theca cell production of A4 (149). High leptin may also interfere with oocyte development and contribute to infertility in PCOS (150). A meta-analysis of leptin levels in people with PCOS found that leptin levels were significantly higher in individuals with PCOS when compared to controls (148). However, when separating obese and non-obese groups, the obese PCOS group still had significantly higher leptin levels than the obese controls but the non-obese PCOS group compared to non-obese controls did not (148). The meta-analysis also found a strong positive correlation between leptin and HOMA-IR, and a weaker positive correlation between leptin and BMI (148). Therefore, the increased leptin in PCOS may be secondary to obesity and hyperinsulinemia (151). There was also a negative correlation between leptin and testosterone, possibly because testosterone may suppress leptin synthesis (148). It is also possible that leptin positively correlates with A4 in non-obese individuals with PCOS (152). Additionally, there appears to be a relationship between leptin and prolactin as increased prolactin may influence increased leptin levels (131) and leptin may raise prolactin levels (138).

There may be relationships between PCOS adipose tissue and the reproductive hormones. In PCOS, there is a decrease in LH pulse amplitude with increasing BMI (1). In a study of 192 women, estradiol levels were lower in overweight and obese women with PCOS than in normal weight women with or without PCOS (41). In a study of 105 women, women with PCOS appeared to have a higher waist-to-hip ratio than BMI matched controls (142). No other differences in anthropometric variables or abdominal adipose tissue volume and distribution were significant (142). It is therefore unlikely that insulin resistance in PCOS is strongly associated with increased visceral and abdominal fat (142).

**Figure 13:**
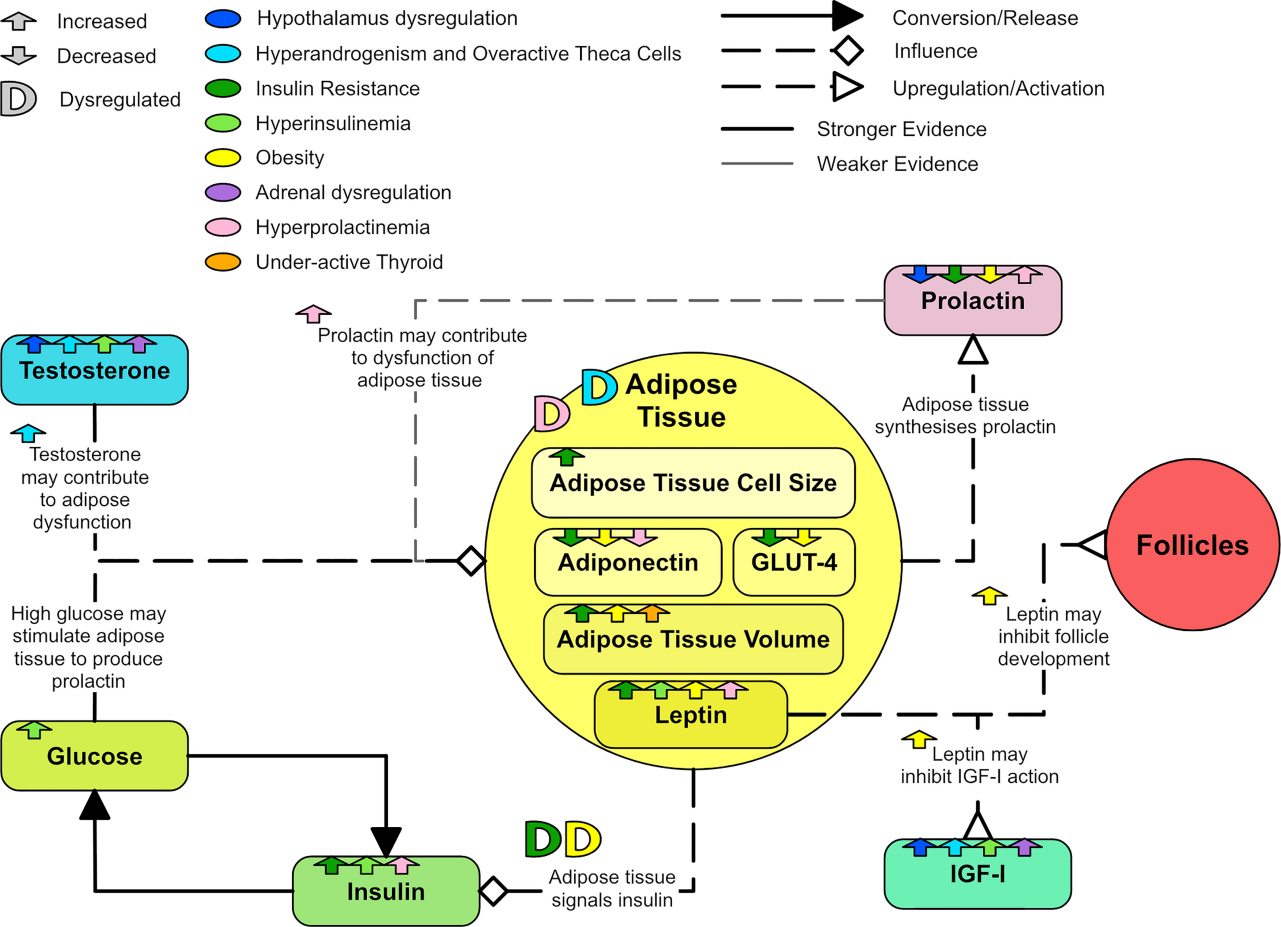
A diagram showing the dysregulation of adipose tissue in PCOS.

It is possible that many of the abnormalities related to adipose tissue in women with PCOS could be secondary to hyperandrogenism (99). An *in vitro* study of human pre-adipocytes found that testosterone caused a time and concentration dependent 50% reduction in lipolysis in subcutaneous fat cells (153). Subcutaneous adipose cells pre-treated with testosterone showed significantly impaired glucose uptake and insulin response *in vitro* (154). Hyperandrogenism has been linked to a decrease in adipose LDL receptor mRNA expression (111). Prolactin may induce changes in adipose tissue, inhibit lipid activity in adipose tissue and decrease adiponectin serum concentration (131). **Figure 13** shows how adipose tissue can be dysregulated in PCOS.

### 3.7 Gut hormones and gut microbiota in PCOS

The gut is the largest endocrine organ in the body, producing multiple hormones that have important signaling roles in multiple metabolic pathways (155). The gut contains the largest number of bacteria and the greatest number of species compared to other areas of the body (156). Gut microbiota (GM) also have many important signaling and metabolic functions (156). Unsurprisingly, the gut is emerging as an important organ in the hormonal signaling pathways associated with PCOS.

Gastric inhibitory polypeptide (GIP) and glucagon-like peptide 1 (GLP-1) are gut hormones known as incretins. Incretins influence insulin secretion from the pancreas in response to ingested food (157). In healthy individuals, GIP and GLP-1 account for 60-75% of insulin secretion following glucose ingestion (158). Fasting GIP appears elevated in individuals with PCOS compared to controls (159, 160). Suppressing GIP has been shown to alleviate insulin resistance (161). GLP-1 increases insulin sensitivity, cognitive function and satiety (161). During a glucose tolerance test, late phase active GLP-1 levels have been shown to be decreased in lean individuals with PCOS compared with controls (160). One study of obese individuals with PCOS found that GLP-1 was lower in prediabetic participants compared to those with normal glucose tolerance (162). They also found correlations between decreased GLP-1 response, increased visceral adipose tissue and decreased insulin sensitivity as measured by the oral glucose insulin sensitivity index (162). Treatment with metformin appears to increase GIP and GLP-1 (163). Exploration of using GLP-1 receptor antagonists as a treatment for PCOS has shown that GLP-1 receptor antagonists appear effective in weight reduction and decreasing HOMA-IR (157).

The human gut holds many different communities of GM. There are four prime phylum, with *Firmicutes* and *Bacteroidetes* making up approximately 90%, and *Actinobacteria* and *Proteobacterium* making up approximately 10%, of all GM (164). Many factors can lead to changes in GM, including age, antibiotics and diet, with diet being one of the most influential factors (165). GM have a range of functions and researchers have often found relationships between different GM and obesity, diabetes, and liver function (166). Some GM are also associated with increased androgens and decreased estrogens (166). More recently, the relationship between PCOS and GM is being explored, with most researchers agreeing that people with PCOS have different GM communities when compared to healthy controls (167). However, at this early stage, the specific differences in the GM of an individual with PCOS are difficult to conclude, with many studies finding contradictory results.

Many studies report a decrease in alpha-diversity (168–171) and/or beta-diversity (169, 172) in PCOS groups when compared to control groups (167, 173, 174). Diversity is often negatively correlated with obesity (169, 172), androgens (169–171, 175) and markers of metabolic issues (169). There is an acknowledged relationship between decreased GM diversity and metabolic dysfunction (167, 170). Some studies also reported a decreased richness in GM in PCOS groups compared to controls, with the obese PCOS group being the least enriched (176, 177).

One of the most consistent findings throughout the literature was an increase in the *Bacteroides* genus (belonging to the *Bacteroidetes* phylum) within PCOS groups (169, 170, 176–179). *Bacteroides* are pro-inflammatory bacteria that can reduce activation of the gut-brain axis control of insulin through a reduction of GLP-1 (178). Increased *Bacteroides* can lead to an increase in branched chain amino acids (BCAAs) (179) and a reduction in bile acids (166). Increased BCAAs are associated with T2DM (165) and are a predictor in IR and diabetes (180). Bile acids emulsify fats, promote digestion, increase the absorption rate of fat-soluble substances, affect lipid metabolism, regulate glucose metabolism and enhance insulin sensitivity (165, 180). *Bacteroides* also produce lipopolysaccharide (LPS) that have links to chronic inflammation, obesity and insulin resistance (177). LPS is linked to signaling that promotes tumor necrosis factor alpha (TNF-α) and interleukin 6 (IL-6) (167), two pro-inflammatory cytokines. Both may be increased in PCOS and higher in IR PCOS (151, 178), leading to a chronic inflammatory state and IR (165). TNF-α increase can also lead to increased intestinal permeability (167). One study found that the increase in *Bacteroides* were greater in IR PCOS when compared to non-IR PCOS (178). Another found the increase was greater in obese PCOS when compared to non-obese PCOS (177). A positive correlation between a *Bacteroides* species and LH levels has also been reported (176).

The *Gammaproteobacteria* class (of the *Proteobacteria* phylum) has been consistently observed to be elevated in individuals with PCOS compared to controls (175–177, 179). At a genus level, an increase in *Escherichia* (176, 177, 179) and/or *Shigella* (177, 179) were often mentioned. *Proteobacteria* have been found to be higher in those with T2DM, metabolic syndrome and inflammatory bowel disease, while *Gammaproteobacteria* is specifically higher in those individuals with NAFLD (175). One study found that the species of *Escherichia* they found increased in PCOS also positively correlated with insulin and negatively correlated with good cholesterol (176).

Some studies found that *Actinobacteria* were increased in obese or PCOS groups when compared to healthy controls (171, 172, 177). At a genus level *Atopobium* (172), *Scardovia* (172), *Collinsella* (177) and *Slackia* (177) were increased in obese groups when compared to non-obese groups, while *Rothia* was found increased in an obese PCOS group when compared to controls (177). Results regarding the various species and families in the *Firmicutes* phylum were inconsistent (171, 174, 177, 179).

**Figure 14:**
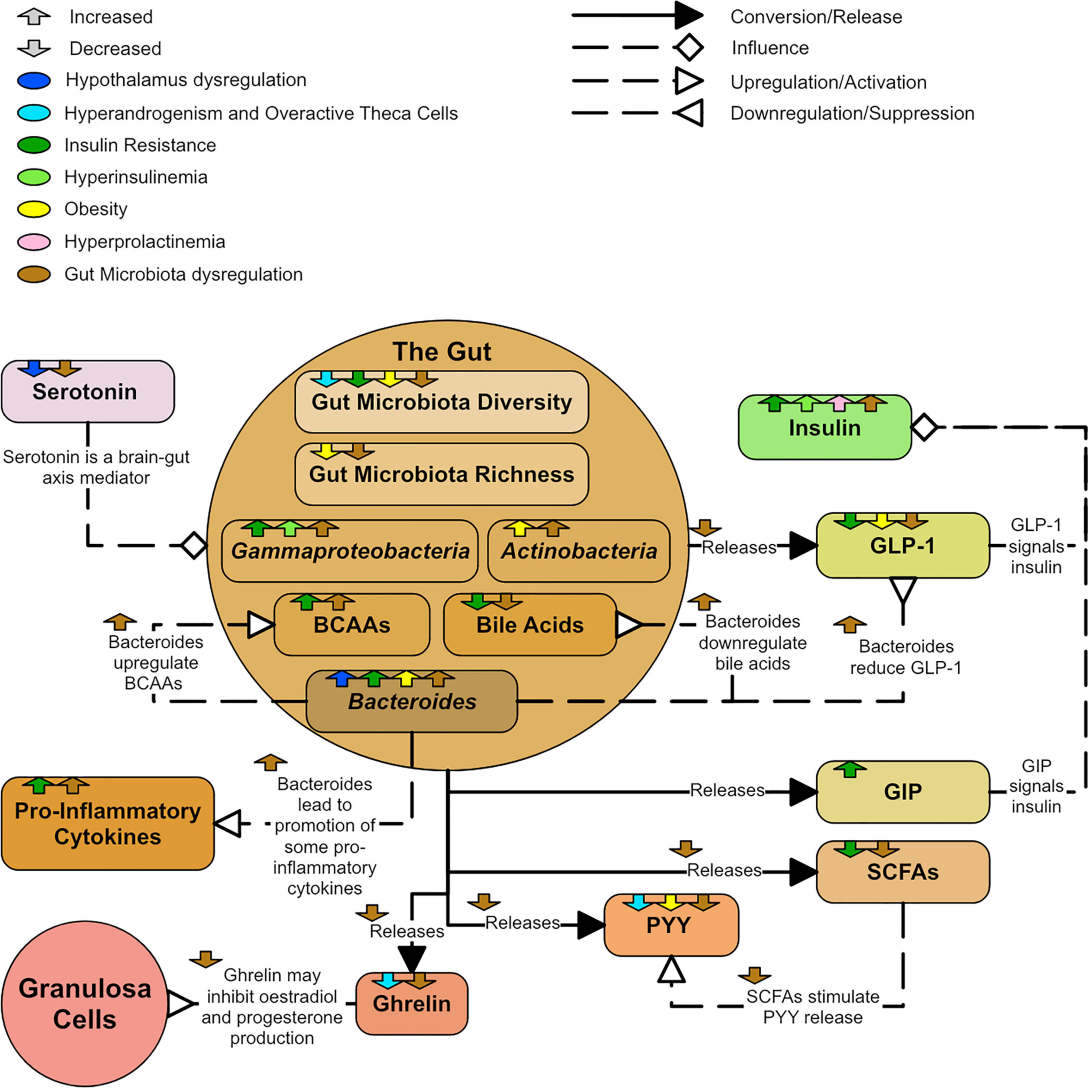
A diagram showing the dysregulation of the gut in PCOS.

There are many potential pathways between dysregulated GM and PCOS (**Figure 14**). GM are involved in the production of short chain fatty acids (SCFAs), which protect intestinal barrier integrity, promote insulin secretion and improve metabolism (180). There are many mediators of the brain-gut axis, such as serotonin, ghrelin, and peptide YY (PYY). Some studies reported a decrease in ghrelin and PYY in PCOS groups compared to controls (177, 179). Ghrelin and PPY negatively correlate with testosterone and liver enzymes and are lower in more severe PCOS phenotypes (177). PYY generally appears lower in obese individuals (161) and SCFAs are known to stimulate PYY release (165). PYY also promotes energy absorption in the intestinal tract (165). Ghrelin may inhibit estradiol and progesterone production (181). Levels of serotonin, which appears to be involved in appetite regulation and psychological wellbeing in PCOS, appears significantly lower in PCOS groups and obese controls compared to non-obese controls (177). Serotonin may also inhibit GnRH and LH secretion (182).

## 4 Unifying model

**Figure 15:**
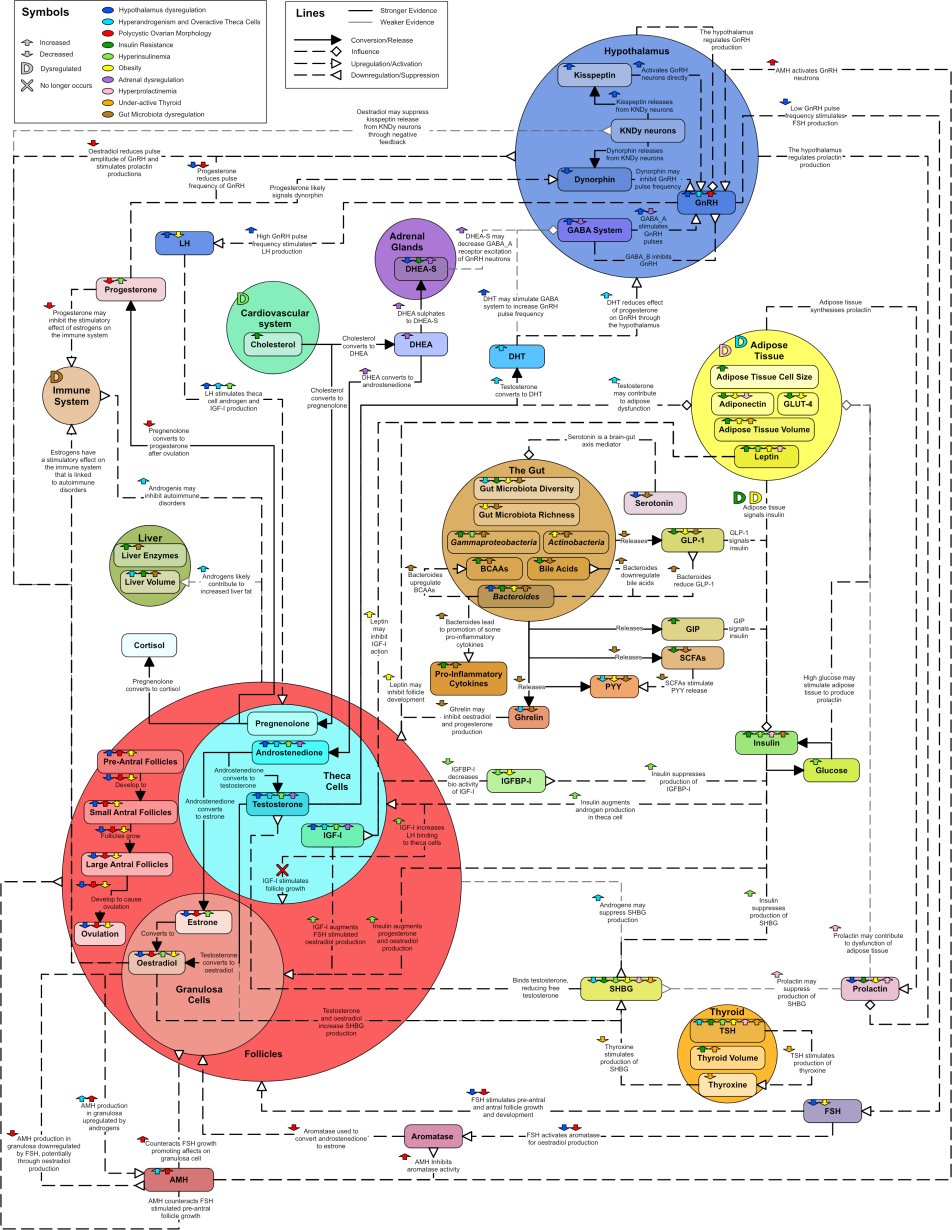
Unifying diagram of PCOS reproductive and metabolic dysregulation. Colored arrows are used to show how various features of the diagram are increased or decreased by possible types of dysregulation or correlations between features. A colored ‘D’ is used to indicate when a feature is dysregulated by something. A colored ‘X’ indicates a relationship that no longer occurs due to a dysregulation. The colors used to represent types of dysregulation are as follows: dark blue for dysregulation in the hypothalamus, light blue for overactive theca cells and hyperandrogenism, red for PCOM, dark green for insulin resistance, light green for hyperinsulinemia, yellow for obesity, purple for adrenal dysregulation, pink for hyperprolactinemia, orange for an underactive thyroid and brown for gut microbiota dysregulation. Solid line arrows between features are used to indicated conversion or release. Dashed lines with diamond arrowhead connections are used to indicate influence. Dashed lines with arrowheads are used to indicate upregulation and dashed lines with reverse arrowheads are used to represent downregulation. Thin grey lines indicate weaker evidence.

A diagram was created to describe the major hormonal signaling pathways associated with endocrine and metabolic dysregulation seen in PCOS (**Figure 15**). The diagram aims to capture the different aspects of PCOS and suggest why PCOS may present with distinct phenotypes. In particular, colored arrows have been included to show how hormones and processes may increase or decrease, with different colors representing potentially different etiologies or phenotypes. They were also used to show known correlations between hormones. While many hormones can be produced and circulate in many different parts of the body only the most significant or pertinent hormone interactions and pathways are noted within the diagram. The coloring of the text boxes and circles are for aesthetic purposes.

## 5 Discussion and conclusions

PCOS is an overlapping collection of phenotypes, associated with a complex hormonal dysregulation, which is not fully understood. In **Figure 15** it can be observed that any of the featured dysregulation types can cause PCOS like symptoms due to the interconnectivity of the endocrine system. With specific blood test results to inform the process, **Figure 15** could be used as a basis to enable patient, or phenotype specific models of PCOS. Such models may enable estimation of the likely efficacy of hormone treatments on individuals.

Due to the heterogeneous nature of PCOS, it is important for researchers to specify the pathological features of each PCOS group they are studying. Without this specification, PCOS studies can appear contradictory. Currently, most researchers use phenotypes that can be derived from the Rotterdam criteria (**Table 1**) to group women with PCOS. However, the Rotterdam phenotypes do not directly consider the metabolic dysfunctions underlying PCOS. Other researchers sometimes classify a subset of PCOS called ‘lean’ PCOS in an attempt to capture women who do not present with metabolic effects. However, 75% of lean women with PCOS are still insulin resistant (7) and a large literature review on lean PCOS indicated that the clinical presentation is comparable to that of overweight/obese PCOS, but that evidence is limited (183). Some research suggests splitting PCOS based on the presence of reproductive issues and/or metabolic issues (184, 185) and possibly even renaming the metabolic side as a different disorder (185). However, as **Figure 15** implies, the reproductive and metabolic aspects of PCOS are interwoven.

**Table 1:** Rotterdam phenotypes (1).

Phenotype	Common Name	Evidence of Hyperandrogenism	Evidence of Oligo-ovulation or Anovulation	Ultrasonic Evidence of PCOM
A	Classic PCOS	+	+	+
B	Essential NIH Criteria	+	+	–
C	Ovulatory PCOS	+	–	+
D	Non-Hyperandrogenic PCOS	–	+	+

PCOS, polycystic ovary syndrome; NIH criteria, National Institutes of Health 1990 conference diagnostic criteria; PCOM, polycystic ovarian morphology (such as increased follicle density or ovary volume).

Many clinicians stress the importance of tailored (individualized) clinical care when treating PCOS (186). However, patient-specific treatment requires an ability to describe the phenotype of the patient, in as much detail as possible. With such an approach, rather than treating the most prominent symptoms, the treatment of PCOS phenotypes may be best considered as managing both the symptoms and the most prominent underlying dysregulations. It is possible that **Figure 15** could elucidate the underlying phenotype of PCOS patients by augmenting the interpretation of laboratory test results. This ability to capture the patient phenotype in a more holistic way (dual focus on both hormonal pathophysiology and presenting symptoms), may reduce the clinical and personal burden of treatment for this complex condition.

## Author contributions

JR and PD conceived the idea. RE and JR researched and wrote the first draft. RE made the diagrams. RE, PD, HL, RC, JR, and KM reviewed and edited. All authors contributed to the article and approved the submitted version.

## Funding

This study was supported by the UC Accelerator Scholarship, University of Canterbury and the Canterbury Medical Research Foundation Summer Scholarship.

## Conflict of interest

The authors declare that the research was conducted in the absence of any commercial or financial relationships that could be construed as a potential conflict of interest.

## Publisher’s note

All claims expressed in this article are solely those of the authors and do not necessarily represent those of their affiliated organizations, or those of the publisher, the editors and the reviewers. Any product that may be evaluated in this article, or claim that may be made by its manufacturer, is not guaranteed or endorsed by the publisher.
